# TiO_2_ Nanorod Array for Betavoltaic Cells: Performance Validation and Enhancement with Electron Beam and ^63^Ni Irradiations

**DOI:** 10.3390/nano15120923

**Published:** 2025-06-14

**Authors:** Sijie Li, Tongxin Jiang, Yu Cao, Wendi Zhao, Haisheng San, Xue Li, Lifeng Zhang, Xin Li

**Affiliations:** 1 China Institute of Atomic Energy, Beijing 102413, Chinalixue@ciae.ac.cn (X.L.);; 2 Pen-Tung Sah Institute of Micro-Nano Science and Technology, Xiamen University, Xiamen 361005, China; jiangtongxin@stu.xmu.edu.cn (T.J.); 33520231153275@stu.xmu.edu.cn (Y.C.); 33520221153301@stu.xmu.edu.cn (W.Z.); 3 Shenzhen Research Institute, Xiamen University, Shenzhen 518000, China

**Keywords:** betavoltaic, TiO_2_ nanorod array, electron beam, NiO_x_

## Abstract

The growing demand for reliable micropower sources in extreme environments has accelerated the development of betavoltaic cells (BV cells) with high energy conversion efficiency and superior radiation resistance. This study demonstrates an advanced BV cell architecture utilizing three-dimensional TiO_2_ nanorod arrays (TNRAs) integrated with a NiO_x_ hole transport layer (HTL). Monte Carlo simulations were employed to optimize the cell design and determine the fabrication parameters for growing TNRAs on FTO substrates via hydrothermal synthesis. The performance evaluation employed both electron beam (2.36 × 10^9^ e/cm^2^·s) and ^63^Ni (3.4 mCi/cm^2^) irradiation methods. The simulation results revealed optimal energy deposition characteristics, with ~96% of β-particle energy effectively absorbed within the 2 μm thick FTO/TNRA/NiO_x_/Au structure. The NiO_x_-incorporated device achieved an energy conversion efficiency of 4.84%, with a short-circuit current of 119.9 nA, an open-circuit voltage of 324.2 mV, and a maximum power output of 24.0 nW, representing a 3.76-fold enhancement over HTL-free devices. Radioactive source testing confirmed stable power generation and linear efficiency scaling, validating electron beam irradiation as an effective accelerated testing methodology for BV cell research.

## 1. Introduction

With the rapid development of wireless sensing networks for harsh environments (deep sea, deep space, and polar and desert regions), the demand for micropower sources has increased significantly. In comparison with conventional cells (chemical cells, fuel cells, and solar cells), betavoltaic (BV) cells are considered ideal power sources for harsh environment applications owing to their advantages, such as long lifetime [1,2], maintenance-free design [3,4], insensitivity to the environment [5,6], and high energy density [7,8].

BV cells can directly convert the energy of β-particles emitted by radioisotopes into electricity through the BV effect. This process is achieved by collecting the charge carriers that are generated when β-particles excite semiconductor materials, enabling current amplification and energy conversion [9,10,11]. Conventional BV cells predominantly utilize monocrystalline silicon or porous silicon materials, fabricated through microelectronic processing techniques to form planar *p-n* junction or 3D *p-n* junction structures [12,13]. However, the silicon-based BV cells generally exhibit low energy conversion efficiency (*ECE*) and short lifespans, which are primarily attributed to the limitations in both the bandgap and radiation resistance of semiconductors [14,15]. Olsen et al. demonstrated that the *ECE* of BV cells increases with semiconductor bandgap [16]. Theoretical calculations suggest that BV cells utilizing wide-bandgap semiconductors can achieve a maximum *ECE* of ~32% [17]. In comparison with low-bandgap silicon, wide-bandgap semiconductors enable BV cells to achieve a higher open-circuit voltage (*V*_oc_) due to the excitation of higher-energy electrons. Furthermore, *p-n* junctions fabricated from wide-bandgap semiconductors exhibit lower reverse saturation current, which enhances *V*_oc_ and consequently improves both power output and *ECE*. Wide-bandgap semiconductors typically possess superior radiation resistance due to their high atomic density, resulting in enhanced operational stability and longer device lifetimes [18,19]. In the previous decades, the commercial wide-bandgap materials, such as SiC, 4H-SiC, and GaN, have been employed in the fabrication of BV cells, but the reported values of *ECE* are in the order of 6% [20,21,22,23,24]. To increase the output power of BV cells, multiple BV cells are stacked to generate microwatt-level output power [25]. Despite research efforts, no significant progress in *ECE* enhancement has been achieved, with current prototypes failing to satisfy practical engineering applications. In recent years, one-dimensional (1-D) nanostructured wide-bandgap semiconductors (e.g., free-standing TiO_2_ nanorod arrays (TNRAs)) have been extensively employed in solar cells, energy storage batteries, photocatalysis, and optoelectronic devices [26,27,28,29]. It has been demonstrated that the large specific surface area of TNRAs can significantly increase the loading capacity of radioactive sources, thereby enhancing the collection efficiency of β-particles. Moreover, the highly ordered 1-D structures facilitate the separation and transport of β-generated carriers, effectively reducing carrier recombination probabilities.

In this work, a TNRA-based BV cell was designed and fabricated. NiO_x_ was used to form the hole transfer layer (HTL) to incorporate with TNRAs for enhanced BV effect. Monte Carlo (MC) simulations were utilized to optimize the design, and the performance validation of the BV cells was verified using electron beam (EB) and ^63^Ni irradiations on the devices.

## 2. Simulation and Preparation of Devices

### 2.1. Monte Carlo Modeling and Simulation

**Figure 1:**
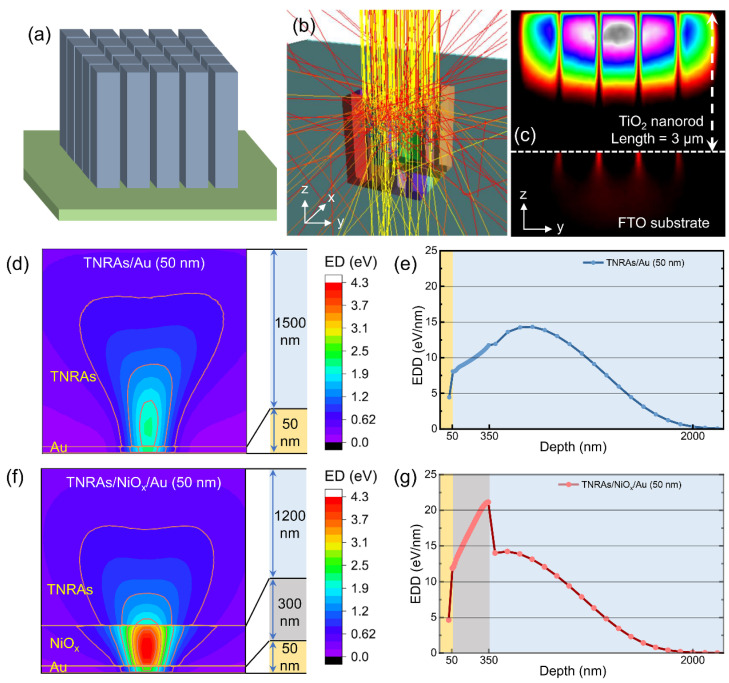
MC modeling and simulation of ECSs under β-radiations with an average kinetic energy of 17.4 keV. (**a**) TNRA model for simulation; (**b**) trajectory lines of β-particles in TNRAs; (**c**) ED distribution of β-particles in *z-y* plane of TNRAs; (**d**) depth-dependent ED distributions and (**e**) dependence of EDD on depth in TNRA/Au structures; (**f**) depth-dependent ED distributions and (**g**) dependence of EDD on depth in TNRA/NiO_x_/Au structures.

The interaction between β-particles and energy conversion materials was studied using the MC simulation via Casino and MCNP5, to simulate the transport pathways and energy transfer mechanisms of β-particles emitted from the ^63^Ni radiation source within BV cells. The incident electron energy was set to 17.4 keV, which is equivalent to the average decay energy of the ^63^Ni radiation source. Meanwhile, the direction collimation design was carried out for the incident electron beam in the simulation process, so that all electrons were incident to the material in the normal direction. There are fewer backscattered electrons on the material surface and a higher energy deposition (ED) value in the material from the simulation results, compared with the actual Ni-63 isotope source that emits particles at a 4π Angle. Simulating the ED of electrons in materials mainly involves the atomic number and density of the materials (TiO_2_: 4.23 g/cm^3^, NiO_x_: 6.67 g/cm^3^, Au: 19.3 g/cm^3^). Figure 1a shows the structural schematic diagram of TNRAs. The size of the nanorods was set as 300 nm in diameter and 3 μm in height with 50 nm spacing between the nanorods, which were arranged in a quadrilateral symmetry according to the actual surface morphology of the grown TNRAs using the hydrothermal synthesis technology (see experimental section). As the β-particles impinge on the surface of TNRAs, multiple collisions occur between the β-particles and TiO_2_ nanorods, generating scattering trajectory lines of β-particles, as shown in Figure 1b. During the collision process, the kinetic energy of β-particles was transferred to the lattices of TiO_2_, thereby forming an ED distribution in TNRAs, as shown Figure 1c. It can be seen that the energy deposition density (EDD) reaches the maximum at a depth of ~500 nm, and the maximum depth of energy deposition reaches ~2 μm. In addition, some β-particles pass through the gaps between the nanorods and are deposited onto the FTO substrate, which does not occur in actual devices owing to the close-packed interconnection at the base regions of TNRAs. In this study, two energy conversion structures (ECSs) based on TNRAs were used for simulation, namely the FTO/TNRA (3 μm)/Au (50 nm) structure and the FTO/TNRA (3 μm)/NiO_x_ (300 nm)/Au (50 nm) structure. The trajectories of the β-particles in two types of ECSs are shown in Supplementary Materials S1. Figure 1d,f show the depth-dependent ED distributions in the TNRA/Au and TNRA/NiOx/Au structures, respectively. The contour lines mark the isopleths where the energy has decayed to different percentages of its initial value. Extracting the simulation data from the surface normal direction at the incident point, the depth-dependent EDD of the two types of ECSs can be obtained, as shown in Figure 1e,g, and the corresponding simulation results are shown in Table 1. It can be observed that the deposited energy of β-particles in the Au film is relatively low, with approximately 96% of the energy being effectively deposited in the two ECSs without regard for the backscattering of β-particles in the Au surface and the self-absorption effect of the radioactive sources. It is worth noting that the NiO_x_ layer has a strong absorption effect on β-particles compared to the TiO_2_ layer. Owing to the large difference in the material density, there is an abrupt numerical change in EDD at the interface between the two layers.

### 2.2. Preparation of BV Cells

#### 2.2.1. Growth of FTO/TNRAs

**Figure 2:**
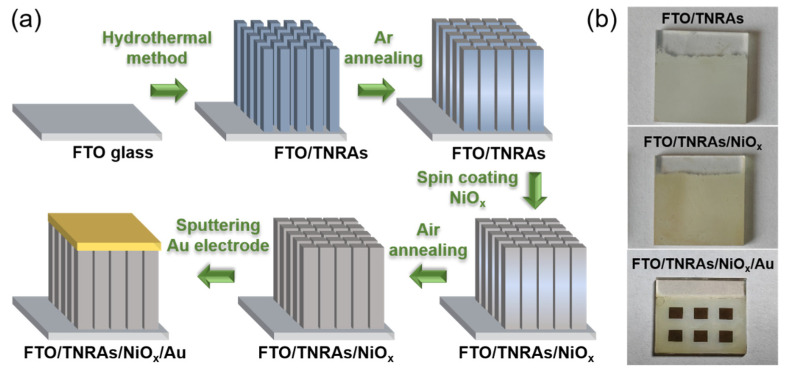
(**a**) Schematic fabrication process flow of FTO/TNRA/NiO_x_/Au; (**b**) photos of the FTO/TNRA, FTO/TNRA/NiO_x_, and FTO/TNRA/NiO_x_/Au samples.

The TNRAs were grown on an F-doped SnO_2_ transparent conductive glass substrate (glass/FTO, 20 mm × 20 mm × 2.2 mm, ≤14 Ω/square, Nanotech, Suzhou, China) using a one-step hydrothermal method and subsequent Ar annealing treatment, as shown in Figure 2a. The conductive glass/FTO substrates were ultrasonically cleaned in an isopropanol, ethanol, and acetone (1:1:1) mixed solution for 1 h, and then an aqueous precursor solution was synthesized for the growth of TNRAs on the glass/FTO substrates, consisting of 0.4 mL of tetrabutyl titanate (C_16_H_36_O_4_Ti), 12 mL of hydrochloric acid (HCl, 36.5 wt%), and 12 mL of deionized water. Next, the aqueous precursor solution and the glass/FTO substrates were transferred into a Teflon-lined stainless steel autoclave and heated at 150 °C for 20 h. Finally, the as-prepared pristine TNRA samples were cleaned and rinsed with deionized water and ethanol solution. Next, the samples were annealed in Ar atmospheres at 450 °C for 1 h to form a crystalline FTO/TNRA sample, as shown in Figure 2b.

#### 2.2.2. Coating of NiO_x_ Film on FTO/TNRAs

First, NiO_x_ nanoparticles were dispersed in deionized water at a mass ratio of 1:4 to form a homogeneous suspension. Next, the NiO_x_ suspension was dropped onto TNRA substrates. A spin-coating method using 2000 rpm/s for 30 s was employed for film formation. Finally, the samples were annealed on a hot plate at 135 °C for 15 min to form FTO/TNRA/NiO_x_ samples.

#### 2.2.3. Sputtering of Au Electrodes on FTO/TNRAs/NiO_x_

A square acrylic plate with dimensions of 20 mm × 20 mm was used for the shadow mask, in which six 3 mm × 3 mm square apertures were fabricated to form the Au electrode windows. The shadow mask was covered on the FTO/TNRA/NiO_x_ and FTO/TNRA samples, and then the magnetron sputtering (EXPLORER-14, Denton, TX, USA) of Au (50 nm) was performed to form the Au/NiO_x_/TNRA/FTO and Au/TNRA/FTO structures.

### 2.3. Material Characterization and Device Measurements

The morphology of samples was examined by field emission scanning electron microscopy (FE-SEM, SUPRA55-SAPPHIRE, Zeiss Microscopy, Oberkochen, Germany) with an energy-dispersive X-ray spectroscopy (EDS, Zeiss Microscopy, Oberkochen, Germany) facility. The crystal structure was characterized by X-ray diffraction analysis (XRD, Rigaku Ultima IV, Rigaku Corporation, Tokyo, Japan). An electron beam generator (China Institute of Atomic Energy, Beijing, China) was used for electron beam irradiation. The composition of materials was analyzed by X-ray photoelectron spectroscopy (XPS, K-Alpha, Thermo Fisher, San Diego, CA, USA). The electrical parameters of the BV cells were measured using a digital source meter (2450, Keithley, Cleveland, OH, USA) in a dark Faraday shielding box.

## 3. Results and Discussion

**Figure 3:**
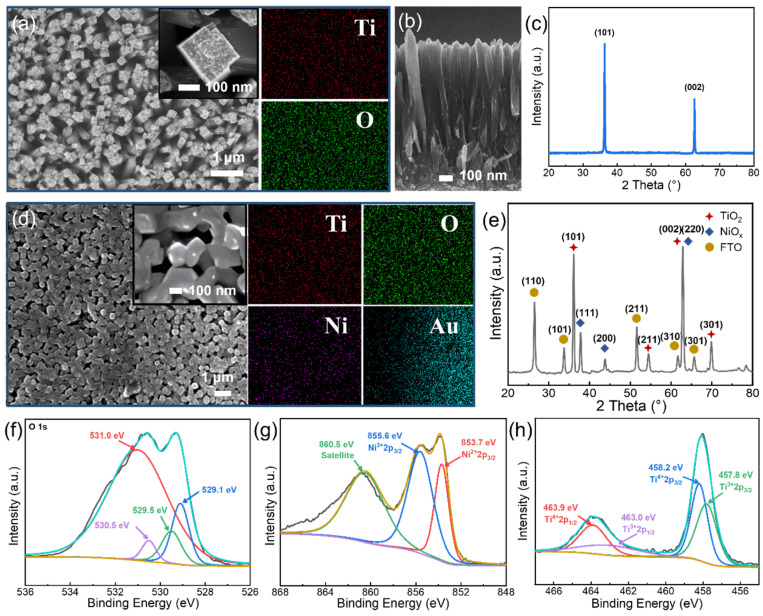
(**a**) Top view image of TNRA and EDS mappings; (**b**) cross-section view image of TNRAs; (**c**) XRD spectra of the TNRAs; (**d**) top view image of TNRA/NiO_x_/Au and EDS mappings; (**e**) XRD spectra of the FTO/TNRAs/NiO_x_/Au; experimental and fitted (**f**) O 1s, (**g**) Ni 2p, (**h**) Ti 2p XPS spectra of TNRAs and NiO_x_.

Figure 3a reveals that the TNRAs are composed of high-density arrays of TiO_2_ nanorods. As shown in the inset of Figure 3a, these nanorods display a distinct tetragonal columnar morphology. The nanorods have diameters ranging from 150 to 200 nm, with a spacing of 40 to 200 nm between adjacent rods and a vertical height of approximately 2 μm (as shown in Figure 3b). These nanorod arrays form a porous network, where the interconnected gaps provide a large specific surface area, which is beneficial for the capture of incident β-particles. According to the XRD phase analysis in Figure 3c, the prepared TNRAs present a typical tetragonal rutile structure, with characteristic diffraction peaks at 2*θ* = 36.1° and 62.8°, corresponding to the (101) and (002) crystal planes of rutile TiO_2_. In comparison with the XRD analysis for the NiO_x_-coated TNRA film in Figure 3e, characteristic peaks corresponding to the cubic phase NiO_x_ are observed at 37.3°, 43.3°, and 62.9°, which correspond to the (111), (200), and (220) crystallographic planes, respectively. It is noticeable that the NiO_x_ diffraction peaks exhibit relatively low intensities, indicating a reduced long-range lattice order. This can be attributed to the lower annealing temperature employed. The surface morphology of the FTO/TNRA/NiO_x_/Au sample is shown in Figure 3d, revealing that NiO_x_ has been uniformly coated on the TNRA surface. The boundary of the Au-coated region can be clearly identified through the EDS spectrum analysis. To investigate the valence states of the elements in the sample, XPS measurements were performed. As shown in Figure 3g, the O 1s spectra follow a Gaussian–Lorentzian distribution. In the XPS analysis of NiO_x_ (Figure 3g), the Ni 2p_3/2_ spectrum shows three characteristic peaks. The peak at 853.7 eV corresponds to Ni^2+^ ions in the Ni-O octahedral coordination structure, the peak at 855.6 eV is attributed to Ni^3+^ ions formed by Ni^2+^ vacancies, and the peak at 860.5 eV is a satellite peak. According to the results in Figure 3h, the characteristic peaks at 457.8 eV, 458.2 eV, 463.0 eV, and 463.9 eV correspond to the binding energies of Ti^3+^ 2p_3/2_, Ti^4+^ 2p_3/2_, Ti^3+^ 2p_1/2_, and Ti^4+^ 2p_1/2_, respectively [30,31]. Material characterization analysis reveals that the Ar annealing process successfully induces oxygen vacancy (OV) defects. Following Ar annealing treatment at 450 °C, the TNRA lattice undergoes directional reconstruction, resulting in the deintercalation of lattice oxygen in the form of O_2_, leading to a high concentration of OVs. With thermodynamic driving, the adjacent Ti^4+^ ions undergo self-reduction to form Ti^3+^ defect centers. Notably, NiO_x_, serving as an HTL, inherently contains a large number of oxygen vacancies. The OV and Ti^3+^ defects can form a shallow donor level (electron traps) just below the conduction band, which are considered to not only contribute to the enhanced carrier density that is beneficial to the charge carrier transport, but also to largely affect the charge separation process by the trapping of electrons [32].

**Figure 4:**
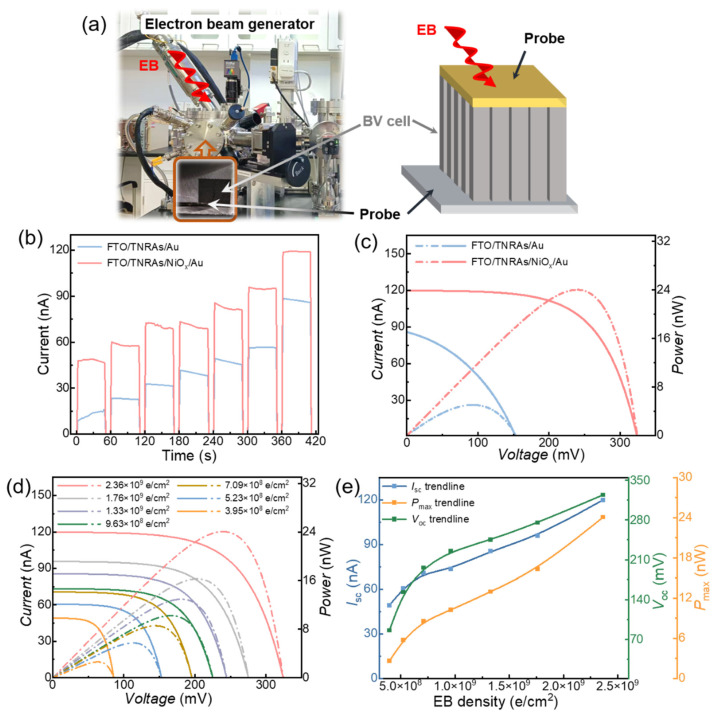
(**a**) Photos of EB generator and tested sample in EB vacuum chamber. Inset is a schematic diagram of BV cell under EB irradiation; (**b**) EB response characteristics of BV cells with and without NiO_x_ layer under EB irradiations with varying fluences; (**c**) *I*-*V* and *P*-*V* curves of BV cells with and without NiO_x_ layer under EB irradiations of 2.36 × 10^9^ e/cm^2^·s; (**d**) *I*-*V* and *P*-*V* curves of BV cell with FTO/TNRA/NiO_x_/Au structure under EB irradiations at varying fluences; (**e**) dependences of *I*_sc_, *V*_oc_, and *P*_max_ on fluences.

Figure 4a shows the photos of the EB generator and tested sample in the EB vacuum chamber. A schematic diagram of the BV cell under EB irradiation exhibits the test probes and incident direction of EB. Two types of BV cells based on FTO/TNRA/Au and FTO/TNRA/NiO_x_/Au structures were irradiated by an EB with electron energy of 17.4 keV. The testing method for the EB density is described in Supplementary Materials S2. Figure 4b shows a comparison of EB response characteristics of two types of BV cells under on/off-switched EB irradiation with step-increasing EB densities. All the devices exhibit a step-increasing and reproducible pulse response of EB current. Furthermore, with the same EB density, the FTO/TNRA/NiO_x_/Au device can achieve the maximum EB response current. The NiO_x_ as the HTL plays an important role in enhancing the response current of the device. It can be observed that the response current of the devices with NiO_x_ was increased by around one-fold compared to that without NiO_x_. It is suggested that the conduction band offset (CBO = 2.5 eV) of NiO_x_ acts as an effective energy barrier to block the excess excited electrons at the TiO_2_ from crossing the interface to the Au electrode. The CBO barrier effectively inhibits the carrier recombination, which is beneficial for the increase in EB response current. Figure 4c shows the current–voltage (*I-V*) and power–voltage (*P-V*) curves of two BV cells under EB irradiation of 2.36 × 10^9^ e/cm^2^·s. The short-circuit current (*I*_sc_) and open-circuit voltage (*V*_oc_) of the BV cell based on the FTO/TNRA/NiO_x_/Au structure are approximately 1.4 times higher than that based on FTO/TNRA/Au. Meanwhile, the maximum output power (*P*_max_) achieved 24.0 nW with a fill factor (*FF*) of 0.62, which was increased by 3.76 times compared to the device without NiO_x_. The *ECE* of the BV cell based on the optimum structure was calculated to be 4.84%. Figure 4d displays the *I*-*V* and *P*-*V* characteristics of the optimized BV cell under EB irradiation at varying fluences. The electrical output parameters are summarized in Supplementary Materials S3. Under identical incident electron energy conditions, the *FF* of the BV cell remains constant at 0.62, independent of the EB fluence. The electrical performance characteristics, including *I*_sc_, *V*_oc_, and *P*_max_, as functions of EB fluence are shown in Figure 4e. It can be observed that *I*_sc_, *V*_oc_, and *P*_max_ increase nonlinearly with EB fluence when the fluence is below 7.5 × 10^8^ e/cm^2^·s. However, once this threshold is exceeded, these parameters exhibit a linear increase with further increases in EB fluence. This indicates that the *ECE* of the devices maintains linear efficiency scaling when the EB density exceeds the threshold value.

**Figure 5:**
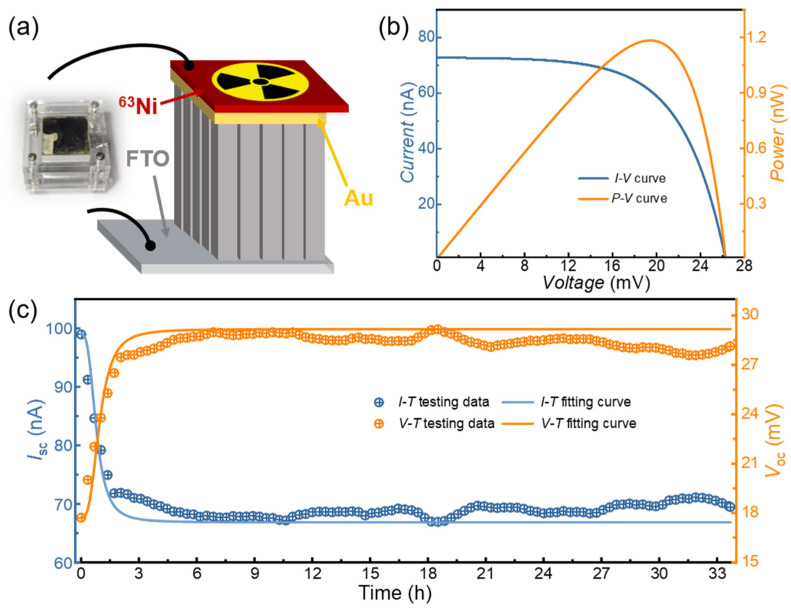
(**a**) Schematic diagram and actual photo of BV cell loaded with ^63^Ni radioactive source; (**b**) *I*-*V* and *P*-*V* characteristics and (**c**) *I*-*T* and *V*-*T* characteristics of BV cells based on FTO/TNRAs/NiO_x_/Au/^63^Ni.

Figure 5a shows a schematic diagram of the BV cell loaded with the ^63^Ni radioactive source, and the actual photo of the BV cell is also exhibited. The size of the ^63^Ni source is 10 mm × 10 mm, and the activity density is 3.4 mCi/cm^2^. The radiation source was assembled on the surface of the Au electrode, which was clamped together with the device by a fixture to ensure close contact between the ^63^Ni source and the electrode. Figure 5b shows the *I-V* and *P-V* curves of the BV cell based on the FTO/TNRA/NiO_x_/Au/^63^Ni structure. The measured results demonstrated that the *P*_max_ reached 1.18 nW with *I*_sc_ = 72.7 nA and *V*_oc_ = 26.3 mV, and the overall *ECE* of the BV cell was 3.74%. The measured results show a slight difference from those obtained using EB irradiation. This can be attributed to the divergent electron emission from the radiation source, which has a broad energy spectrum, in contrast to highly collimated electrons with consistent kinetic energy. It validates the effectiveness through the utilization of EB irradiation as an alternative to actual radioactive sources. The performance of BV cells can be systematically assessed through the experimental measurement employing equivalent EB irradiation. A comparison of recent study results in BV cell research is presented in Table 2. To evaluate the operational stability of BV cells, long-term electrical performance metrics (*I*_sc_ and *V*_oc_) of the BV cells were systematically measured during a 33.5 h continuous monitoring test. As shown in Figure 5c, the *I*_sc_ exhibited rapid degradation during the initial 2 h period, decreasing from 98.9 nA to ~70 nA. Subsequently, it entered a relatively stable phase with minor fluctuations around this 70 nA level throughout the remaining measurement period. In contrast, the *V*_oc_ displayed an opposite trend; it showed a significant increase during the first 2 h, rising from 17.7 mV to 28.5 mV, and subsequently stabilized with minor fluctuations around the 28.5 mV level over the subsequent measurement period. These measured results suggest that the FTO/TNRA/NiO_x_/Au structure exhibits a distinct capacitive behavior, characterized by rapid discharging under short-circuit conditions and charging under open-circuit voltage measurements.

**Figure 6:**
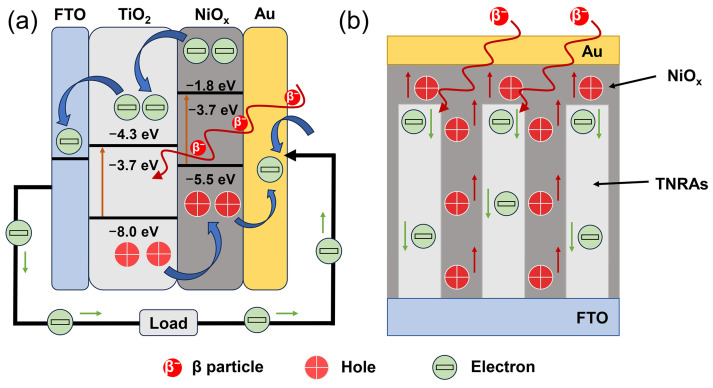
(**a**) Schematic diagram of the energy band and working principle of BV cells based on FTO/TNRA/NiO_x_/Au structure; (**b**) schematic charge transfer processes of β-excited carriers in BV cells.

Figure 6a shows the schematic energy band diagram and operational mechanisms of BV cells based on the FTO/TNRA/NiO_x_/Au structure. In this system, high-energy β-particles penetrate the Au layer (~50 nm) and impinge on the TNRA/NiO_x_ heterostructure, generating a large amount of EHPs via impact ionization. During this process, the β-excited electrons migrate from the conduction bands of both NiO_x_ and TiO_2_ to the FTO conductive glass and subsequently reach the Au cathode through an external circuit. Simultaneously, the β-excited holes transfer from the valence bands of both TiO_2_ and NiO_x_ to the Au electrode. Consequently, a complete carrier transport cycle is established. The NiO_x_/TiO_2_ heterojunction can effectively separate the EHPs and suppress their recombination in the cycle. The NiO_x_/TiO_2_ heterojunction exhibits a type-II band alignment with a 2.5 eV potential barrier in the NiO_x_ conduction band relative to TiO_2_, effectively blocking electron transfer from β-excited TiO_2_ to the NiO_x_ HTL. Figure 6b shows a schematic illustration of the charge transfer processes of β-excited carriers in the BV cell. The NiO_x_ HTL coats the surface of the TNRA structure, resulting in a formation of a large specific area of the 3-D network of the NiO_x_/TiO_2_ heterojunction. With the space field in the junction, the holes migrate to the NiO_x_ HTL and then to the Au electrode while the electrons transfer to the FTO substrate along the TiO_2_ nanorods. The orthogonal separation routes of the electrons and holes effectively suppress the recombination of EHPs.

## 4. Conclusions

In conclusion, this study presents a BV cell based on 3-D wide-bandgap semiconductive nanostructure TNRAs incorporated with a NiO_x_ HTL for enhancing both energy conversion efficiency and radiation degradation. MC simulations were utilized to optimize the design of BV cells, and TNRAs were grown on the FTO substrate using a hydrothermally synthesized method. The performance validation of the BV cells was verified using both EB and ^63^Ni irradiations on devices, respectively. The simulated results show that the maximum energy deposition depth reached ~2 μm, with approximately 96% of the energy being effectively deposited in the FTO/TNRA/NiO_x_/Au structure. Under electron beam radiation (2.36 × 10^9^ e/cm^2^·s), the device demonstrated an *ECE* of 4.84% with an *I*_sc_ of 119.9 nA, *V*_oc_ of 324.2 mV, and *P*_max_ of 24.0 nW, which is a 3.76-fold enhancement over the device without the NiO_x_ HTL. Furthermore, a ^63^Ni source was assembled in the BV cell, and a stable output in electrical power was achieved with an *ECE* of 3.74%, validating electron beam irradiation as an effective accelerated evaluation method. The working principle of BV cells indicates that the NiO_x_ HTL coated on the surface of TNRAs enables an orthogonal separation route of electrons and holes in nanorods, effectively suppressing the recombination of EHPs and thus enhancing the *ECE* of BV cells.

## Figures and Tables

**Table 1:** EDR of β-particles in various material layers of ECSs.

	Au (keV)	NiO_x_ (keV)	TNRAs (keV)	ED Efficiency
TNRAs/Au	0.22	—	16.74	96.2%
TNRAs/NiO_x_/Au	0.23	5.11	11.52	95.6%

**Table 2:** A comparison of BV cells in recent years.

	Radiation Source	Conversion Device	*V*_oc_ (V)	*η* (a.u.)	Ref.
1	^241^Am	Diamond	1.06	1.41%	[33]
2	^63^Ni	ZnO	0.50	3.58%	[18]
3	^147^Pm	Si	0.17	2.30%	[34]
4	^147^Pm	GaN	1.25	2.78%	[35]
5	^237^Np	SiC	1.99	0.88%	[20]
6	He^2+^	GaN	1.13	4.51%	[36]
7	^63^Ni	SiC	0.40	0.28%	[37]
8	^63^Ni	Si	0.26	2.21%	[38]
9	^63^Ni	GaP	1.48	2.68%	[39]
10	^63^Ni/EB	TNRAs	0.026/0.32	3.74%/4.84%	This work

## Data Availability

The raw data supporting the conclusions of this article will be made available by the authors on request.

## Supplementary Materials

The following supporting information can be downloaded at https://www.mdpi.com/article/10.3390/nano15120923/s1, Figure S1: The trajectory of β-particles with an energy of 17.4 keV in the energy conversion material, Figure S2: Calibration of the effective current value under various beam current intensities, Table S1: Effective beam current and electron flux, Table S2: Electrical parameters of BV cells based on FTO/TNRA/NiO_x_/Au structure under EB irradiations at varying fluences.

## Abbreviations

BV	betavoltaic
TNRA	TiO_2_ nanorod array
HTL	hole transport layer
*ECE*	energy conversion efficiency
*V*_oc_	open-circuit voltage
1-D	one-dimensional
MC	Monte Carlo
EB	electron beam
ED	energy deposition
ECS	energy conversion structure
OV	oxygen vacancy
*I-V*	current–voltage
*P-V*	power–voltage
*I*_sc_	short-circuit current
*P*_max_	maximum output power
*FF*	fill factor
EDD	energy deposition density
