# Supplementary material for: TiO2 Nanorod Array for Betavoltaic Cells: Performance Validation and Enhancement with Electron Beam and 63Ni Irradiations
Source: Nanomaterials (Basel). 2025 Jun 14;15(12):923. doi: 10.3390/nano15120923 (PMC12195721; doi:10.3390/nano15120923)
Supplement: Supplementary file 1 [file nanomaterials-15-00923-s001.zip › nanomaterials-3653491-supplementary.pdf]

TiO_2 nanorod array for betavoltaic cells: Performance validation and enhancement with electron-beam and ^{63}Ni irradiations

Sijie Li[1,†], Tongxin Jiang[2,3,†], Yu Cao[2,3], Wendi Zhao[2,3], Haisheng San[2,3,*], Xue Li[1], Lifeng Zhang[1,*], Xin Li[1]

[1] China Institute of Atomic Energy, Beijing 102413, China; lisijie@ciae.ac.cn (S. L.); lixue@ciae.ac.cn (Xue L.); lix@cnncmail.cn (Xin L.)

[2] Pen-Tung Sah Institute of Micro-Nano Science and Technology, Xiamen University, Xiamen 361005, China; jiangtongxin@stu.xmu.edu.cn (T. J.); 33W520231153275@stu.xmu.edu.cn (Y. C.); 33520221153301@stu.xmu.edu.cn (W. Z.)

[3] Shenzhen Research Institute of Xiamen University, Shenzhen 518000, China; jiangtongxin@stu.xmu.edu.cn (T. J.); 33520231153275@stu.xmu.edu.cn (Y. C.); 33520221153301@stu.xmu.edu.cn (W. Z.)

* Correspondence: sanhs@xmu.edu.cn (H. S.); zhanglifeng@ciae.ac.cn (L. Z.)

† Those authors contributed equally to this work.

S1 The trajectory of β particles with an energy of 17.4 keV in the energy conversion material.

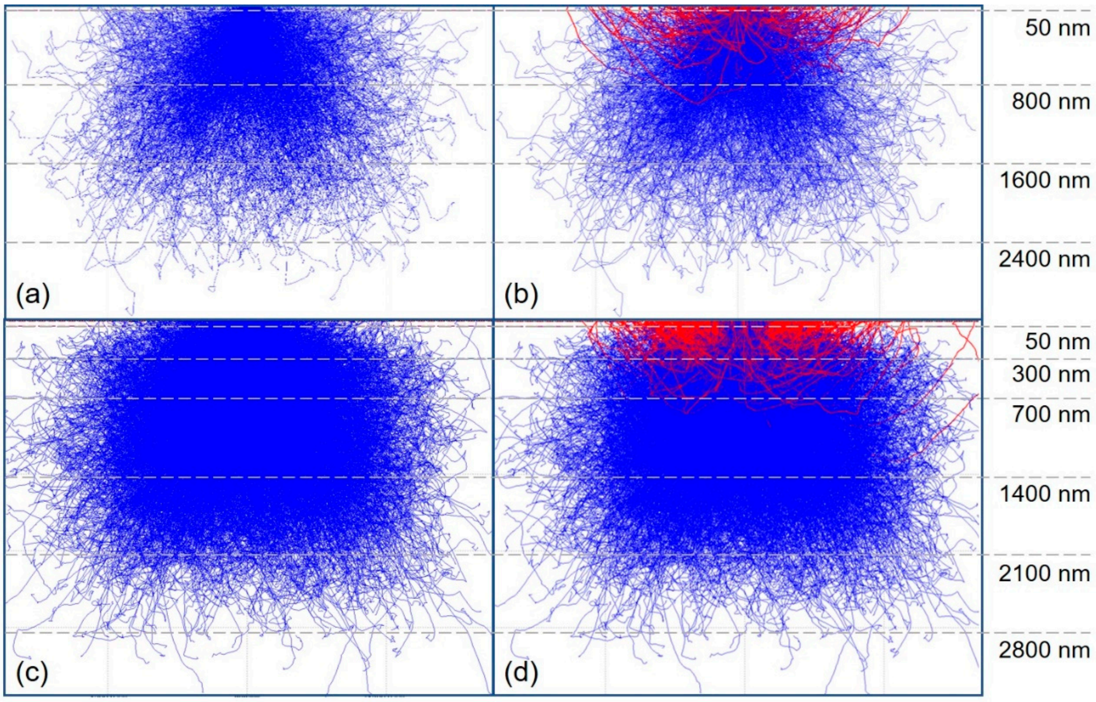

Figure S1 The β particles track in TNRAs/Au (50 nm) (a) without, (b) with backscattering electrons; The β particles track in TNRAs/NiO_x/Au (50 nm) (a) without, (b) with backscattering electrons.

S2 Testing and correction of EB density.

The experiment adopted an independently-built measurement system to test the EB density of 7 groups. The background current value was obtained by using a cantilever tungsten probe, which is the current of the probe-I_{probe}. Subsequently, under the same parameter conditions, a pure copper standard sheet (with dimensions of 10 mm × 10 mm) was placed on the sample stage. The current of the copper sheet was measured- I_{Cu}. The effective current value I_{eff} per unit area was obtained by subtracting the probe current from the copper sheet current, that is:

$$I_{\mathrm{eff}} = I_{\mathrm{Cu}} - I_{\mathrm{probe}}$$

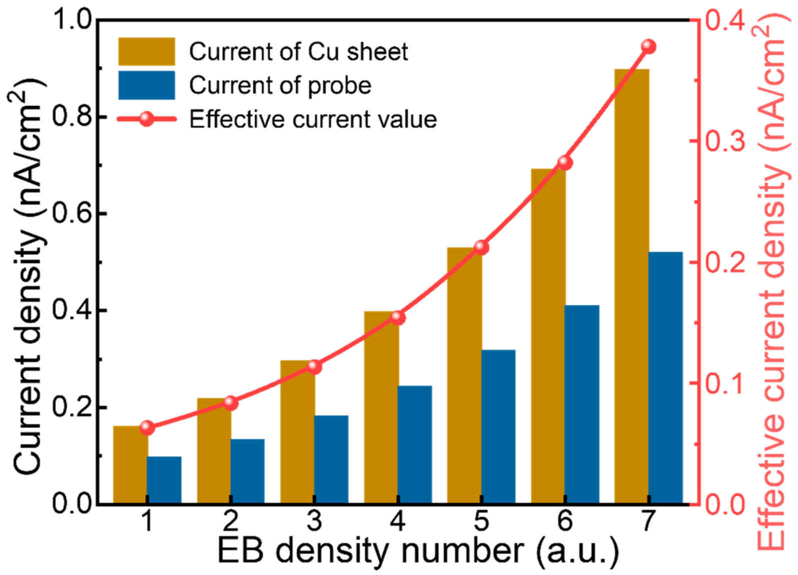

Figure S2 Calibration of the effective current value under various beam current intensities.

The test results are shown in Figure S2. Moreover, the current values per unit area have been converted into the electron flux values per unit area, as presented in Table S2.

Table S1 Effective beam current and electron flux

	Beam 1	Beam 2	Beam 3	Beam 4	Beam 5	Beam 6	Beam 7

I_{eff} (nA/cm^2)	0.0632	0.0836	0.1134	0.154	0.212	0.282	0.378
EB density (e/cm^2·s)	3.95×10^8	5.23×10^8	7.09×10^8	9.63×10^8	1.33×10^9	1.76×10^9	2.36×10^9

S3 The electrical output parameters of optimized BV cell under EB irradiation at varying fluences

Table S2 Electrical parameters of BV cells based on FTO/TNRAs/NiO_x/Au structure under EB irradiations at varying fluences

	3.95×10^8 e/cm²·s	5.23×10^8 e/cm²·s	7.09×10^8 e/cm²·s	9.63×10^8 e/cm²·s	1.33×10^9 e/cm²·s	1.76×10^9 e/cm²·s	2.36×10^9 e/cm²·s
I_{sc}（nA）	49.2	60.6	70.7	73.4	85.5	95.6	119.9
P_{max}（nW）	2.6	5.7	8.6	10.2	12.9	16.3	24.0
V_{oc} (mV)	85.6	152.9	195.7	225.1	244.7	275.2	324.2
FF	0.62	0.62	0.62	0.62	0.62	0.62	0.62
